# Specifying the psychosocial pathways whereby child and adolescent adversity shape adult health outcomes

**DOI:** 10.1017/S003329172200318X

**Published:** 2023-10

**Authors:** Man-Kit Lei, Mark T. Berg, Ronald L. Simons, Steven R. H. Beach

**Affiliations:** 1 Department of Sociology, University of Georgia, Athens, GA, USA; 2 Department of Sociology and Criminology, University of Iowa, Iowa City, IA, USA; 3 Department of Psychology, University of Georgia, Athens, GA, USA

**Keywords:** Biological aging, minority population, prospective longitudinal design, psychosocial maladjustment, self-reported illness, social adversity

## Abstract

**Background:**

Social scientists generally agree that health disparities are produced, at least in part, by adverse social experiences, especially during childhood and adolescence. Building on this research, we use an innovative method to measure early adversity while drawing upon a biopsychosocial perspective on health to formulate a model that specifies indirect pathways whereby childhood and adolescent adversity become biologically embedded and influence adult health.

**Method:**

Using nearly 20 years of longitudinal data from 382 Black Americans, we use repeated-measures latent class analysis (RMLCA) to construct measures of childhood/adolescent adversities and their trajectories. Then, we employ structural equation modeling to examine the direct and indirect effects of childhood/adolescent adversity on health outcomes in adulthood through psychosocial maladjustment.

**Results:**

RMLCA identified two classes for each component of childhood/adolescent adversity across the ages of 10 to 18, suggesting that childhood/adolescent social adversities exhibit a prolonged heterogeneous developmental trajectory. The models controlled for early and adult mental health, sociodemographic and health-related covariates. Psychosocial maladjustment, measured by low self-esteem, depressive and anxiety symptoms, and lack of self-control, mediated the relationship between childhood/adolescent adversity, especially parental hostility, racial discrimination, and socioeconomic class, and both self-reported illness and blood-based accelerated biological aging (with proportion mediation ranging from 8.22% to 79.03%).

**Conclusion:**

The results support a biopsychosocial model of health and provide further evidence that, among Black Americans, early life social environmental experiences, especially parenting, financial stress, and racial discrimination, are associated with adult health profiles, and furthermore, psychosocial mechanisms mediate this association.

## Introduction

Childhood and adolescent social adversities in the form of poverty, abuse, community violence, and racism are stressors experienced by many Black Americans (Hayward & Gorman, 2004; Umberson, Thomeer, Williams, Thomas, & Liu, 2016). Though some youth seem more resilient to childhood or adolescent adversity than others, for most youth, early adversity appears to have enduring associations with various health-related problems in adulthood (Miller, Chen, & Parker, 2011). This is particularly true for Black Americans, who are more likely to face multiple adversities (Suglia et al., 2018) and experience a more significant burden of illness resulting from increased biological wear and tear (Geronimus, 2013). One potential mediator between social adversity and adult health among Black Americans may be lasting effects on psychosocial maladjustment.

In line with the biopsychosocial model of health (Clark, Anderson, Clark, & Williams, 1999), personal psychosocial maladjustments may partially explain how social stress gets ‘under the skin’ to affect adult health outcomes. These processes reflect a collection of interrelated psychological capacities, maladjustments, mood impairments, and distorted beliefs – e.g., depressive and anxiety symptoms (DAS), lack of self-control, and low self-esteem – known to reduce an individual's ability to cope with adversity and, in turn, negatively affect health outcomes (see Taylor, Way, and Seeman, 2011). Early life social experiences are especially crucial to health trajectories as they likely operate as schematic frameworks through which later experiences are filtered and upon which new lessons are layered (Lei, Brody, & Beach, 2022). The literature suggests that childhood/adolescent social adversity presages poor health in large measure indirectly through impaired psychosocial processes (i.e. Winning, Glymour, McCormick, Gilsanz, and Kubzansky, 2016). The present study extends this work in several respects.

First, self-reported measures of health, while widely utilized in research on the social determinants of health, likely partly reflect individuals' emotional and psychological states at the time of assessment rather than objective health conditions (Berg, Lei, Beach, Simons, & Simons, 2020; Harris & Schorpp, 2018). To minimize the self-report biases, Turner (2010) suggested simultaneously considering subjective and objective measures of health outcomes. Recently, biomarkers have been used to measure objective health status. However, most individual biomarkers capture risk associated with a single disease – e.g., glycated hemoglobin A1c (HbA1c) as a biomarker of diabetes risk. Though such specificity of single item biomarker information can be useful, each typically has only a small influence in improving the prediction of mortality and morbidity (Goldman, Glei, & Weinstein, 2017). Thus, researchers have argued there is a need for new tools that provide a comprehensive index of health status (Horvath & Raj, 2018; Simons et al., 2019, 2021a). Peter et al. (2015) identified 1497 sites where the level of gene expression (amount of mRNA) is correlated with age; this mRNA index is known as a ‘biological clock’ in that it assesses how much older or younger a person is (in years) on a biological scale than their chronological age. Studies have reported that this and similar aging clocks are robust predictors of cardiovascular disease, metabolic functioning, and chronic illnesses (Renson et al., 2020); all of which suggests that biological aging involves a multitude of changes in gene expression that presage chronic illness. The current study examines a subjective indicator of perceived health alongside an mRNA index of biological aging.

Second, many studies have used the adverse childhood experience (ACE) score to measure early life social adversity, summing across family abuse, neglect, and household dysfunction to identify cumulative risk (see McLaughlin and Sheridan, 2016). However, this type of measure does not capture adversities present across neighborhood, economic, and broader social domains (Alexander, Entwisle, & Olson, 2014; Umberson et al., 2016). Indeed, it is likely the case that many types of adversity not typically assessed by ACEs are more harmful to health than others. For example, past studies have reported that persistent exposure to discrimination in early life affects the health of Black Americans above and beyond other social adversities (Simons et al., 2021b). To address this issue, we focus on four components of social adversities across different life domains. This approach allows us to examine the role of specific types of adversities that may be particularly relevant to many Black Americans.

Further, a review of previous studies of childhood adversity (e.g. Friedman, Montez, Sheehan, Guenewald, & Seeman, 2015; Suglia et al., 2018) indicates considerable heterogeneity in the experiences of early adversities across time. Most empirical research to date explores adversities assessed at a single point in the life course or examines adversities assessed retrospectively. For example, young adult respondents in the National Longitudinal Study of Adolescent to Adult Health (Add Health) study were asked whether they had experienced adversities before the age of 18. The present study contributes to the literature by measuring components of childhood/adolescent adversity using prospective reports. Using repeated-measures latent class analysis (RMLCA), we determine whether each adversity is best characterized by multiple, distinct trajectories or by a single trajectory. Specifically, we hypothesized that the four components of childhood/adolescent adversity including parental hostility, neighborhood crime, racial discrimination, and socioeconomic (SES) status risk, are identified by one or more trajectory classes from ages 10 to 18. Building on the allostatic load model (Juster, McEwen, & Lupien, 2010) and the biological weathering hypothesis (Forde, Crookes, Suglia, & Demmer, 2019), we hypothesized that compared to low exposure to adversities across time, repeated and frequent exposure to adversity in childhood and adolescence would degrade bodily systems and trigger psychosocial maladjustment processes. These processes combine to influence self-reported illness and accelerated biological aging in adulthood.

Finally, the measurement of adversities is a known and important methodological challenge. Just recently, for instance, the American Heart Association (AHA) released a scientific statement expressing caution about research on of the linkages between social adversity and heart health because most of the work is cross-sectional and based on *retrospective* reports of childhood experiences (see arguments in Suglia et al., 2018: e20). For instance, the Behavioral Risk Factor Surveillance System ACE module developed by the U.S. Centers for Disease Control & Prevention (CDC) asked adult respondents to recall events before 18 years of age (Merrick, Ford, Ports, & Guinn, 2018). However, given that retrospective measures ask participants to recall experiences that, in many instances, occurred years earlier, these measures are known to be vulnerable to the limitations of forgotten memories and selective recall biases (e.g. Baldwin, Reuben, Newbury, & Danese, 2019; Berg *et al*. 2020; Lei, Berg, Simons, Simons, & Beach, 2020). Accordingly, we use longitudinal data collected over 20 years to test the degree to which childhood/adolescent adversities on subjective and objective adult outcomes is mediated by psychosocial maladjustment.

## Methods

### Participants

We tested this hypothesis using data from seven waves of the Family and Community Health Study (FACHS; see online Supplementary Appendix A). At baseline (1997–1998), the FACHS sample consisted of 889 Black American fifth-grade children. Their mean age at the initial wave was 10.56 years. The second, third, fourth, fifth, and sixth waves of data were collected in 1999–2000, 2001–2002, 2004–2005, 2007–2008, and 2011–2012 to capture information when the target children had mean ages of 12.5, 15.7, 18.8, 21.6, and 23.6, respectively. The 2015–2016 data (age 29) collection included blood draws, after excluding samples with poor quality and samples excluded because of no amplification, successful assays for mRNA expression were obtained for 386 individuals. Details regarding mRNA assays are provided in the supplemental materials (online Supplementary Appendix B). In the current study, there were four missing cases for self-reported illness. Analyses are based on 382 respondents (143 men and 239 women). Comparison of those who were not included in this study (*n* = 507) *v.* those who were (*n* = 382) indicated no significant differences in study variables at age 10, suggesting missing completely at random (MCAR) (see online Supplementary Table S1).

### Measures

#### Childhood/adolescent adversity

Given that early adversity often occurs in multiple domains, we assessed four domains of adversities between ages 10 to 18. First of all, *parental hostility* consisted of 14 items (Conger & Elder, 1994) concerning if the primary caregiver directed various hostile and coercive behaviors toward participants (*α* ⩾ 0.80). Then, the five-item *neighborhood crime* scale (Sampson, Raudenbush, & Earls, 1997) asked about the frequency (never = 1, often = 3) of various criminal acts occurring within the target's residential community, including behaviors such as fighting, sexual assault or rape, robbery or mugging, and gang violence (*α* ⩾ 0.75). Third, *racial discrimination* was measured using a four-item scale (Whitbeck, Hoyt, McMorris, Chen, & Stubben, 2001), which assessed the frequency (never = 1, several times = 4) of various discriminatory events experienced by the respondents (*α* ⩾ 0.75). Finally, *SES risk* was assessed by primary caregiver's report on a nine-item index about family financial events and adjustment developed by Conger and Elder (1994) (*α* ⩾ 0.70). These scales have been used in numerous studies and have strong reliability and validity (e.g. Conger *et al*. 1992; Lei, Beach, Simons, & Ye, 2021; Simons *et al*. 2019). The items for these four indicators can be found in the online Supplementary Materials, Appendix C.

#### Self-reported illness symptoms

At age 29, respondents were asked if they had experienced (not experienced = 0, severe symptoms = 3) 18 acute illness symptoms: cough, runny nose, swollen glands, sore throat or fever, headache, stiff or aching muscles and/or joints, fatigue, asthma and/or respiratory allergies, pain in back, neck, or shoulders, urinary problems, constipation, heart-burn and/or indigestion, nausea, diarrhea, dizziness, breathlessness, racing heartbeat, palpitations, or chest pain, and numbness or tingling. The measure has been used in prior research on health and well-being and has strong reliability and validity (e.g. Berg *et al*. 2020; Haugland & Wold, 2001). Items were summed to form an index of self-reported illness (*α* ⩾ 0.90).

#### Accelerated biological aging

Biological aging was measured using the transcriptomic clock developed by Peters et al. (2015) and using algorithms available through an online mRNA aging calculator (https://trap.erasmusmc.nl). Mean blood-based biological age was 29.49 (s.d. = 2.98). Unstandardized residual scores from the regression of biological age on chronological age we calculated to measure accelerated biological aging. These residuals had a mean of zero and represented both positive and negative deviations from chronological age (in years), with positive scores indicating accelerated biological aging.

#### Adult psychosocial maladjustment

At ages 21 to 25, three scales were used to assess adult psychosocial maladjustment including DAS, lack of self-control, and low self-esteem. *DAS* was assessed with a nine-item scale that captured feelings of anxiety and depression (Kessler et al., 1994) (*α* ⩾ 0.80). *Lack of self-control* was assessed using 11 items from Kendall & Wilcox's (1979) control scale (*α* ⩾ 0.70). *Low self-esteem* was evaluated using five items from a revised version of the Rosenberg's Self-Esteem Scale (Rosenberg, Schooler, & Schoenbach, 1989) (*α* ⩾ 0.75). A list of the items used to measure the indicators of maladjustment can be found in online Supplementary Appendix D.

#### Control variables

Analyses included controls for *gender* (male = 1) and health-related covariates. At ages 10 to 18, body mass index (BMI) at ages 10 to 18 was calculated as weight in kilograms divided by height squared in meters, and childhood depression was assessed with the Diagnostic Interview Schedule for Children – Version 4 (DISC–IV; Shaffer et al., 1993). At age 29, *Education* was measured by asking respondents the question: ‘What is the highest level of education you have completed?’ and respondents were asked to report have you been diagnosed by a professional for depression to measure *adult depression*. Further, both *healthy diet* and *exercise* were assessed with two items developed for the current project (see online Supplementary Appendix E).

### Analytic strategy

All analyses were run using M*plus* 8.3. We first conducted RMLCA of the four domains of childhood/adolescent adversity from ages 10 to 18 to identify homogeneous subgroups of respondents in the sample (Muthén, 2008). In RMLCA, class membership is not known *a priori*. Instead, respondents are sorted into classes based on their posterior probability of class membership. Utilizing RMLCA allowed us to assess associations among variables in subpopulations that are hidden in the overall sample and allowed us to model change ‘whatever form it naturally occurs in each latent class’ (Collins & Lanza, 2009: 187). Comparisons of model fit among 1-to-3 class RMLCA were evaluated. The entropy (greater than 0.80) and the Lo–Mendell–Rubin adjusted likelihood ratio (LMR-LRT) determined the better-fitting model.

After identifying the optimal number of latent classes, using path modeling, we first examined the indirect effects of the four components of adversity on health outcomes through low self-esteem, DAS, and lack of self-control separately. Specifically, we tested mediating effects by including one mediator (e.g. low self-esteem) while excluding other mediating indicators (e.g. DAS and lack of self-control). It should be noted that although multiple mediator models with the three mediators included simultaneously can identify relative mediation effects, this comes at the cost of regression coefficients that are suppressed or inflated by multicollinearity if these mediators are highly correlated (Gonzalez & MacKinnon, 2021). Because the three indicators of adult psychosocial maladjustment in the current data set are highly correlated (see online Supplementary Table S2), we could not test the three mediators simultaneously without substantial multicollinearity. After examining the mediators separately, we used structural equation modeling (SEM) to examine the extent to which the formed a latent construct of ‘psychosocial maladjustment’ that mediated childhood/adolescent adversities on the health outcomes. Finally, to test the role of different dimensions of adversity, we estimated a mediating model using each of the four components of childhood/adolescent adversities in place of the summation of the four childhood/adolescent adversities.

To assess the goodness-of-fit of the model, we used Steiger's root-mean-square error of approximation (RMSEA < 0.05) and the comparative fit index (CFI > 0.90). Finally, the 95% confidence interval (CI) was estimated with bias-corrected and accelerated bootstrapping with 1000 resamples was used to assess the significance of indirect effects. The percent of mediation (PM) was calculated by the ratio of the indirect effect to the total effect to provide an effect size of each indirect effect (Preacher & Kelley, 2011).

## Results

### Identifying the construct of each childhood/adolescent adversity

**Fig. 1.**
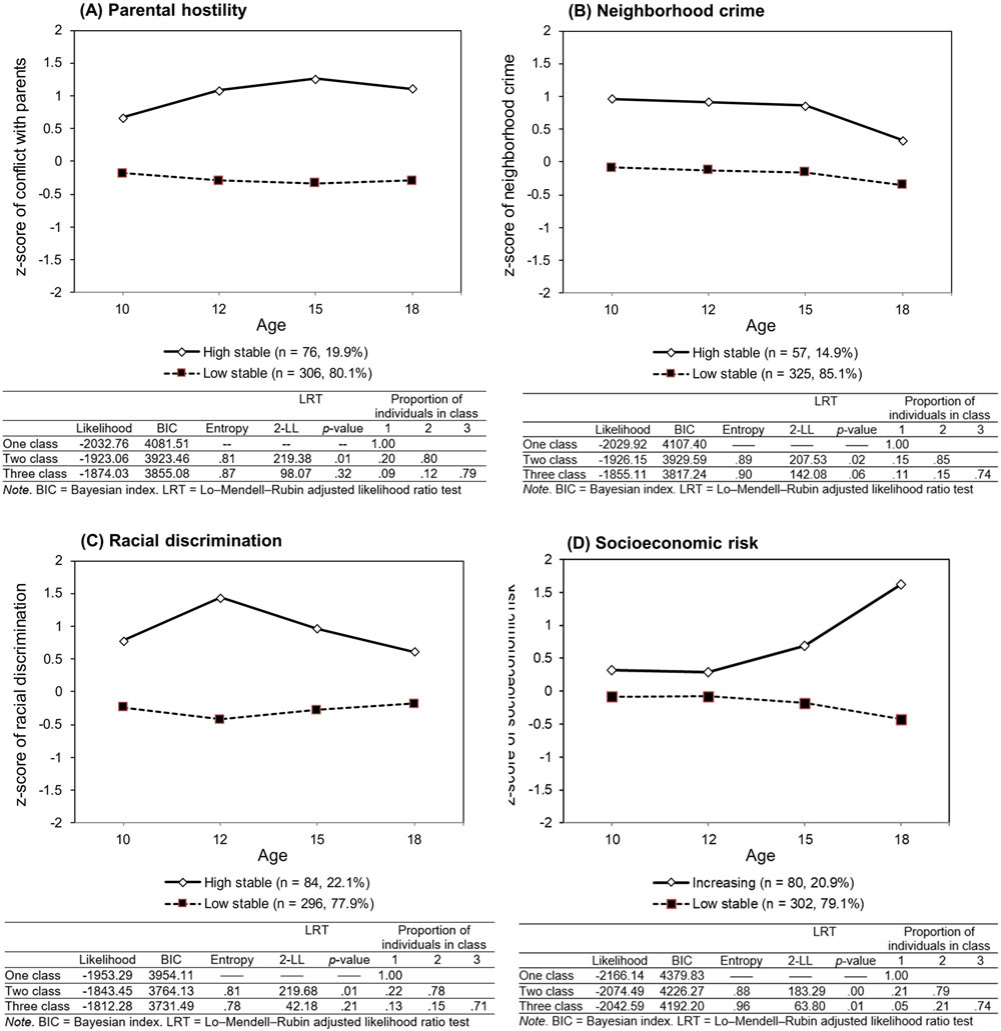
Latent class analysis and model selections of classes of (a) parental hostility, (b) neighborhood crime, (c) racial discrimination, and (d) socioeconomic risk.

Before testing our hypotheses, RMLCA was conducted to identify different classes of trajectories of the four components of childhood/adolescent adversities. Model fit statistics for RMLCA solutions with 1, 2, and 3 latent classes are shown in Fig. 1. The LMR-LRT tests of parental hostility, neighborhood crime, and racial discrimination for the 3-class model were not significant, suggesting that the 2-class model was superior to both 1- and 3-class models. Next, although the 3-class model test of socioeconomic risk was significant, one of the classes for the 3-class model produced an unacceptably small class (*n* = 19, 5%). Further, the 2-class model had an acceptable entropy value, suggesting that the two classes were distinct. Accordingly, for each component, the two-class model was selected. As seen in Fig. 1, the majority of respondents were assigned to a class with a low and stable level of each adversity across time. We refer to this class as ‘low-stable’ = 0. In contrast, the second group showed a relatively high and stable or increasing level of each adversity. We refer to this class as ‘high-stable’ or ‘increasing’ = 1.

The zero-order correlations with mean and standard deviations among the study variables are presented in online Supplementary Table S2. First, high and stable levels of parental hostility and racial discrimination across the ages of 10 to 18 are associated with low self-esteem, DAS, and lack of self-control. Next, increasing socioeconomic risk over time is related to low self-esteem and DAS, but not lack of self-control. A high and stable level of neighborhood crime is correlated with lower self-esteem. Further, low self-esteem, DAS, and lack of self-control, in turn, show significant associations with both self-reported illness and accelerated aging. Because all elements of potential mediation emerged, we proceeded with a formal test of our mediating hypotheses.

### Low self-esteem, DAS, and lack of self-control as mediators

Next, we used path modeling to examine the extent to which the three psychosocial maladjustment indicators mediated the effects of four adversity components on self-reported illness and accelerated aging. As shown in Table 1, the results show significant indirect effects for each adversity through low self-esteem (with P_M_ ranging from 8.22% to 58.06%). Turning to the model for DAS as a mediator, the impacts of parental hostility, racial discrimination, and SES risk on self-reported illness and accelerated aging in adulthood is mediated by DAS (with P_M_ ranging from 12.12% to 79.03%). Finally, lack of self-control significantly mediates the pathways of parental hostility and racial discrimination on health outcomes (with P_M_ ranging from 12.15% to 44.48%). These indirect effects support the hypothesis that childhood/adolescent adversities suffered by Black Americans promote low self-esteem, DAS, and lack of self-control which, in turn, are associated with increased health problems in adulthood.

**Table 1.** Indirect effect models of childhood/adolescent adversity through low self-esteem, DAS, and lack of self-control on self-reported illness and accelerated biological aging

	Self-reported illness (age 29)		Accelerated aging (age 29)	
	Indirect effect		Indirect effect	
Model	coefficient (95% CI)	Percent mediation (*P*_M_)	coefficient (95% CI)	Percent mediation (*P*_M_)
Mediator = Low self-esteem (ages 21–25)				
Parental hostility (ages 10–18)	0.04* (0.02–0.08)	58.06%	0.02 (0.01–0.04)	13.76%
Neighborhood crime (ages 10–18)	0.04 (0.01–0.07)	40.08%	0.01 (0.01–0.04)	8.22%
Racial discrimination (ages 10–18)	0.03 (0.01–07)	15.58%	0.02 (0.01–0.04)	27.27%
Socioeconomic risk (ages 10–18)	0.03 (0.01–0.06)	50.00%	0.01 (0.01–0.04)	14.36%
Mediator = Depressive and anxiety symptoms (ages 21–25)				
Parental hostility (ages 10–18)	0.05 (0.02–0.10)	79.03%	0.02 (0.01–0.04)	12.41%
Neighborhood crime (ages 10–18)	0.02 (−0.01 to 0.05)	ns	0.01 (−0.01 to 0.02)	ns
Racial discrimination (ages 10–18)	0.08 (0.03–0.13)	42.78%	0.03 (0.01–0.06)	50.96%
Socioeconomic risk (ages 10–18)	0.04 (0.01–0.09)	50.49%	0.01 (0.01–0.03)	12.12%
Mediator = Lack of self-control (ages 21–25)				
Parental hostility (ages 10–18)	0.03 (0.01–0.06)	44.48%	0.02 (0.01–0.04)	12.15%
Neighborhood crime (ages 10–18)	0.01 (−0.02 to 0.03)	ns	0.01 (−0.02 to 0.01)	ns
Racial discrimination (ages 10–18)	0.03 (0.01–0.07)	18.71%	0.02 (0.01–0.05)	37.14%
Socioeconomic risk (ages 10–18)	0.02 (−0.01 to 0.05)	ns	0.01 (−0.01 to 0.03)	ns

*Note*: Indirect effect represents the indirect effects of the predictors on self-reported illness and accelerated biological aging via low self-esteem, DAS, and lack of self-control. Percent mediation (*P*_M_) is (Indirect effect ÷ Total effect) × 100. Coefficients are standardized. 95% CI is 95% confidence interval.

**p ⩽ 0.01; *p ⩽ 0.05, †*p* < 0.10 (two-tailed tests).

### The impact of cumulative adversities on health outcomes through latent construct psychosocial maladjustment

**Fig. 2.**
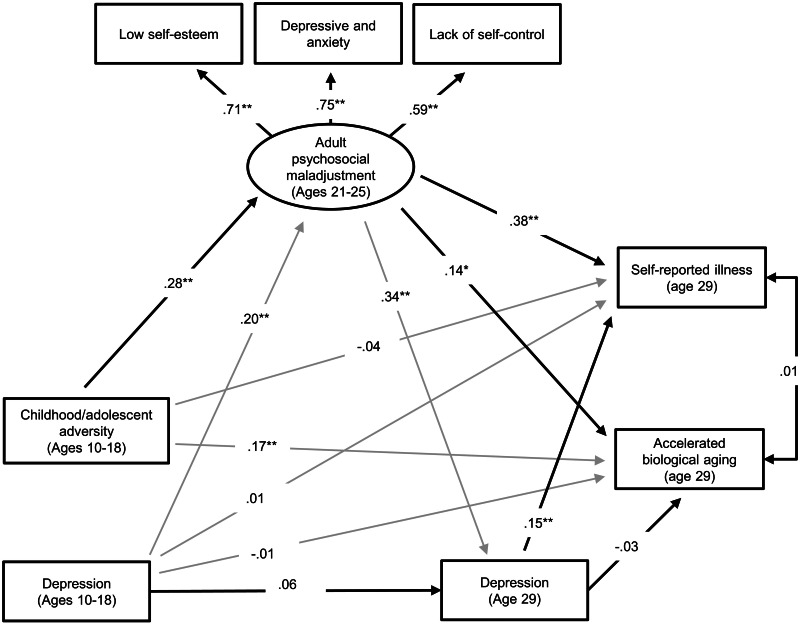
Adult maladjustment mediates the impact of childhood/adolescent adversity on self-reported illness and accelerated biological aging (*N* = 382). *Note:* χ^2^ = 33.14, *df* = 26, *p* = 0.16; CFI = 0.98; RMSEA = 0.03. Values are standardized parameter estimates. Males, education (age 29), body mass index (ages 10–18), healthy diet (age 29), and exercise (age 29) are controlled in these analyses. ^†^ *p* ⩽ 0.10; * *p* ⩽ 0.05; ** *p* ⩽ 0.01 (two-tailed tests).

Given that low self-esteem, DAS, and lack of self-control are highly correlated, we combined these three indicators into a single latent construct labeled psychosocial maladjustment. The measure provides a comprehensive psychosocial measure that accounted for measurement error in the mediator and had greater variance than the individual measures (Gonzalez & MacKinnon, 2021). We began by testing a cumulative risk approach to adversity. The model controls for childhood and adult depression, and other covariates. As shown in Fig. 2, fit indices indicate that the model fits the data well. The model shows that our cumulative measure of childhood/adolescent adversity is associated with adult psychosocial maladjustment, which, in turn, is related to both self-reported illness and accelerated aging in adulthood. Using a bootstrapping technique with 1000 replications, significant indirect effects appear for self-reported illness [IE = 0.121 95% CI (0.05–0.18), P_M_ = 66.9%] and accelerated aging [IE = 0.04, 95% CI (0.01–0.08), P_M_ = 16.1%].

### The influence of parental hostility, neighborhood crime, racial discrimination, and socioeconomic risk on health outcomes through latent construct psychosocial maladjustment

**Fig. 3.**
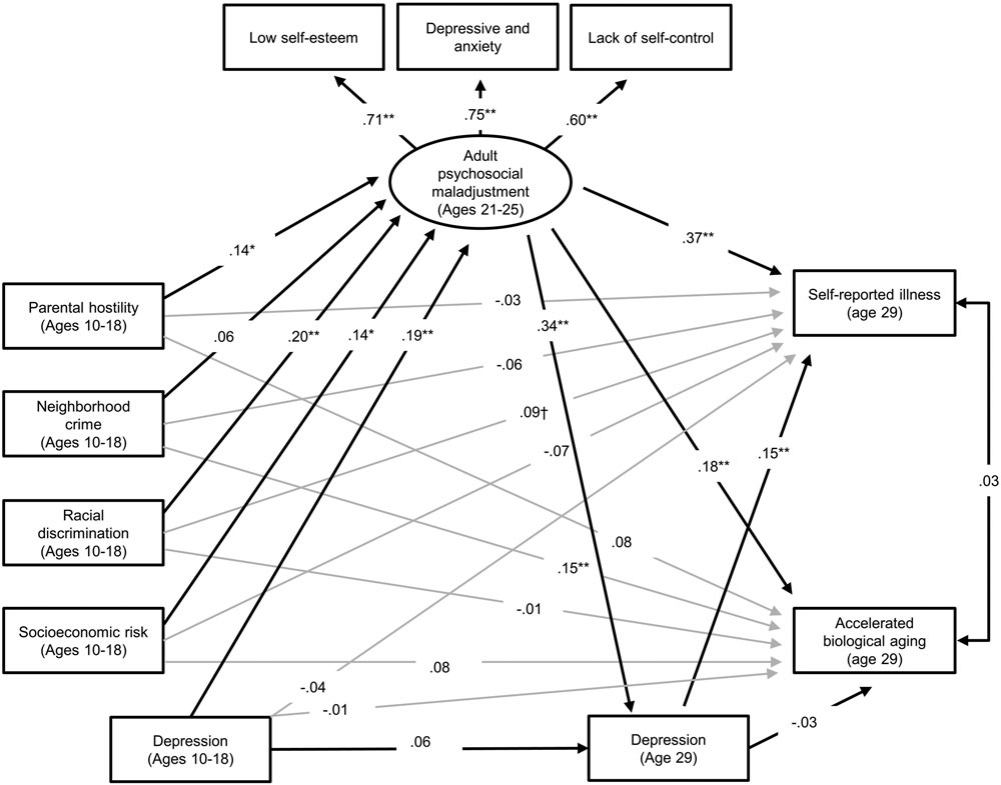
Adult maladjustment mediates the impact of childhood/adolescent adversity (parental hostility, neighborhood crime, racial discrimination, and socioeconomic risk) on self-reported illness and accelerated biological aging (*N* = 382). *Note:* χ^2^ = 46.88, *df* = 35, *p* = 0.09; CFI = 0.97; RMSEA = 0.03. Values are standardized parameter estimates. Males, education (age 29), body mass index (ages 10–18), healthy diet (age 29), and exercise (age 29) are controlled in these analyses. ^†^ *p* ⩽ 0.10; * *p* ⩽ 0.05; ** *p* ⩽ 0.01 (two-tailed tests).

To further examine the independent effects of different dimensions of adversity, we examined each of the four components of adversities simultaneously. As shown in Fig. 3, the various model fit indices indicate that the hypothesized SEM provides a good fit to the data (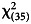 = 46.88, *p* = 0.09; CFI = 0.97; RMSEA = 0.03). Note that the model includes depression at ages 10–18 and 29, demographic characteristics and health-related covariates. The findings show that childhood/adolescent adversities, except for neighborhood crime, are significant predictors of adult psychosocial maladjustment. Psychosocial maladjustment, in turn, is a significant predictor of both self-reported illness and blood-based accelerated aging at age 29. Table 2 shows results from tests of the indirect effects using a bootstrap approach (see Table 2). As shown, parental hostility, racial discrimination, and SES risk had significant indirect effects with self-reported illness and accelerated biological aging (with P_M_ ranging from 16.94% to 72.73%).

**Table 2.** Indirect effect models of childhood/adolescent adversity through adult psychosocial maladjustment (indicators: low self-esteem, DAS, and lack of self-control) on self-reported illness and accelerated biological aging

	Self-reported illness (age 29)		Accelerated aging (age 29)	
	Indirect effect		Indirect effect	
Model	coefficient (95% CI)	Percent mediation (*P*_M_)	coefficient (95% CI)	Percent mediation (*P*_M_)
Mediator = Adult psychosocial maladjustment (ages 21–25)				
Parental hostility (ages 10–18)	0.06** (0.02–0.12)	56.99%	0.02* (0.01–0.06)	16.94%
Neighborhood crime (ages 10–18)	0.02 (−0.02 to 0.07)	ns	0.01 (−0.01 to 0.04)	ns
Racial discrimination (ages 10–18)	0.06** (0.02–0.13)	38.36%	0.02* (0.01–0.06)	72.73%
Socioeconomic risk (ages 10–18)	0.05** (0.02–0.11)	39.10%	0.02* (0.01–0.06)	21.00%

*Note*: Indirect effect represents the indirect effects of the predictors on self-reported illness and accelerated biological aging via the latent variable of adult psychosocial maladjustment (indicators: low self-esteem, DAS, and lack of self-control). Percent mediation (*P*_M_) is (Indirect effect ÷ Total effect) × 100. Coefficients are standardized. 95% CI is 95% confidence interval.

**p ⩽ 0.01; *p ⩽ 0.05, †*p* < 0.10 (two-tailed tests).

### Sensitivity analysis

It is possible that gender differences may play an important role in each pathway. Multiple group analysis in SEM was conducted to test for differences between models for females and males (see online Supplementary Table S3). We compared a model that constrained all paths to be equal between females and males with an alternative model that allowed them to differ. To determine which paths were different, we freed one path in the constrained model at a time and compared it with the constrained model's 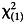. Using this method, only one path showed a significant gender difference (adult psychosocial maladjustment → self-report illness: Δ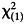 = 12.22, *p* = 0.01). Further, adolescents may not be accurate reporters of their own self-control. To address the robustness of our results, we conducted a sensitivity analysis excluding self-control from the models. The results are similar to those found in Table 2 (see online Supplementary Table S4).

## Discussion

A recent scientific statement from the AHA identified adverse childhood/adolescent experiences as a risk of chronic disease and a significant public health priority especially for Black Americans (Suglia et al., 2018). This omission is vital as adverse conditions such as harsh parenting, SES risk, neighborhood crime, and racial discrimination are more frequently encountered by Black Americans than other racial groups (Umberson et al., 2016). In line with the allostatic load model (Juster et al., 2010), these conditions combine to form a context of adversity that connotes uncertainty, danger, and threat, with the result being enhanced wear and tear on physiological systems and triggering of physiological stress responses in multiple bodily systems. The current study extended this line of research in several ways by: confirming a construct of childhood/adolescent adversity, identifying trajectories of adversity, modeling subjective and objective health outcomes, and examining social-psychological process that may medicate the linkage between early adversity and adult health. Our results revealed that the four domains of social adversities are best characterized by two developmental trajectories: a low-stable group and a high-stable/increasing group. These findings suggest that, in many cases, there is a stable clustering of adverse experiences across the childhood and adolescent periods, and this clustering is associated with differential health risk across individuals.

Consistent with our hypothesis, the four components of childhood/adolescent adversities were associated with self-reported illness and blood-based accelerated aging. Moreover, these linkages between various adversities and adult health remained after controlling for health-promoting behaviors and childhood/early life mental health. That these effects held for accelerated aging is especially noteworthy given that biological aging has been identified as a potential indicator of weathering or dysregulation across various physiological systems (Forde et al., 2019; Renson et al., 2020). Our results also conceptually replicated and extended the existing literature (Argabright et al., 2022; Carter et al., 2019) and suggest that even using multiple sources of data (e.g. self-report and blood assay data) and controlling for adult covariates, persistent exposure to early life adversities is associated with an increased risk of adult health problems.

During the past decade, social scientists have focused on how social environments may ‘get under the skin’ and influence health outcomes (Miller et al., 2011). Various studies identify low self-esteem, DAS, and lack of self-control as potential mediators of the association between the social environment and health-related outcomes. In particular, psychosocial factors related to the self, emotional distress, and pursuit of short-term goals are associated with poor health outcomes (e.g. Taylor et al., 2011). In part, this may reflect the way these mediators affect risky behavior (Lei et al., 2022). Consistent with this view, our results showed that persistent exposure to adversities across the childhood/adolescent life course had significant indirect effects on each health outcome through three variables – low self-esteem, DAS, and lack of self-control.

Given that the three psychosocial mechanisms are highly correlated, we specified psychosocial maladjustment as a latent construct indicated by low self-esteem, DAS, and lack of self-control. Following a cumulative risk approach (McLaughlin & Sheridan, 2016), we employed a summation measure of high/low indicators of the four components of adversities to examine our general model. As expected, we then found that social adversities in childhood/adolescence were associated with blood-derived and self-reported health measures through psychosocial maladjustment. The direct effects of adversities were no longer significant when the latent construct of psychosocial maladjustment was included in the SEM. Our results suggest that psychosocial maladjustment mediates the effect of childhood/adolescent adversity on young adult health.

Among the four components of childhood/adolescent adversities examined in this study, parental hostility, racial discrimination, and SES risk had a significant indirect effect through psychosocial maladjustment. It is worth noting that the absence of an indirect effect of neighborhood crime may be a consequence of Black experiences in a racially stratified society in which disadvantaged neighborhoods are especially affected by unique mechanisms that presage poor health, including low collective efficacy and mistrust. Although there may be multiple pathways from childhood/adolescent adversities to adult health, psychosocial maladjustment represents a critical pathway. Accordingly, this psychosocial pathway could be a target of intervention efforts aimed at reducing the effects of childhood/adolescent adversity on later health.

### Limitations

While our study was able to extend past research in several respects, it is not without shortcomings. First, our sample was limited to Black Americans. Although myriad studies have established that Black Americans are at higher risk than other ethnic groups for health-related outcomes and exposure to early adversity (Umberson et al., 2016), these findings need to be replicated with other racial and ethnical groups. Second, past studies have raised concerns that relationships between early adversity and health may be spurious because child health status may influence adult health outcomes (e.g. Macleod and Smith, 2003). Our study assessed RNA gene expression of aging and self-reported illness at only one point in time (age 29), and thus we were unable to control for earlier health status. Albeit given that high BMI is associated with health problems, we controlled for BMI between ages 10 to 18. Third, we included depression at ages 10–18 and age 29 in the models. However, these mental health measures were limited because of inconsistent measures across time points (e.g. used DISC-IV at ages 10–18 but professional diagnosis at age 29). In addition, parental depression was not included. Given this limitation, we recognize that the findings do not rule out possible additional pathways (e.g. intergenerational transmission of health problems, effects of unmeasured psychopathology, stress propagation, and biological embedding models) (Hammen, Hazel, Brennan, & Najman, 2012). Future studies should focus on multi-generational perspectives concerned with the intergenerational transmission of adversity as well as longitudinal changes in biological aging. Fourth, larger samples may have the power to detect small latent trajectory groups. These small groups may not have a strong theoretical foundation. However, replication in a larger sample will be necessary to determine if this is the case. Finally, like all longitudinal studies, the current study also suffered from sample attrition over time. Although attrition met the assumption of MCAR, replication in a sample with lower attrition would be useful.

### Implications and conclusion

Despite its limitations, the current investigation extended prior research in several respects. Our findings suggest that research should move beyond a single domain, time, and beyond retrospective approaches to measure constellation of adversities based on multiple indicators of health outcomes (e.g. self-reported and blood-based measures), and incorporate psychosocial explanatory processes. It appears that a cluster of interrelated psychosocial processes, including DAS, self-esteem, and self-control, ought to be considered when investigating mechanisms that may account for the relationship between childhood/adolescent social adversities and adult health outcomes. Indeed, in our model, exposure to adversities does not directly influence health but instead contributes to negative emotional and cognitive responses that foster poor adult health outcomes. Our research has important implications. From a theoretical perspective, the findings highlight the importance of constructing integrated models that unite social, psychological, and biological variables, and adopting a biopsychosocial perspective (e.g. the allostatic load model and weathering hypothesis) to enhance scientific knowledge of the social determinants of health. From a methodological perspective, the present study develops an alternative measure of childhood adversity involving multiple dimensions and time effects. From a clinical practice perspective, our results potentially inform the development of clinical practices that focus on improving psychosocial adjustment (i.e. improving self-esteem, reducing negative affect, enhancing self-control) through family and neighborhood resilience mechanisms to mitigate the consequences of early adversities on health outcomes.

## Supplementary material

For supplementary material accompanying this paper visit https://doi.org/10.1017/S003329172200318X. click here to view supplementary material
