# Supplementary material for: Specifying the psychosocial pathways whereby child and adolescent adversity shape adult health outcomes
Source: Psychol Med. 2022 Oct 21;53(13):6027–36. doi: 10.1017/S003329172200318X (PMC10120399; doi:10.1017/S003329172200318X)
Supplement: Supplementary file 1 [file S003329172200318Xsup001.docx]

**Psychological medicine**

**ONLINE SUPPLEMENT**

SPECIFYING THE PSYCHOSOCIAL PATHWAYS WHEREBY

CHILD AND ADOLESCENT ADVERSITY SHAPE ADULT HEALTH OUTCOMES

**Appendix A. Sampling procedures**

The Family and Community Health Study (FACHS) is an ongoing study of several hundred Black American families that was initiated in 1997-1998. The protocol and all study procedures were approved by the University Institutional Review Board. All of the families included a child who was in fifth grade at study inception. Using a stratified random sampling procedure, the sampling strategy was intentionally designed to generate families representing a range of socioeconomic statuses and neighborhood settings (see Gibbons et al., 2004, Simons et al., 2011, 2019). Using 1990 census data, block groups were identified in both Iowa and Georgia in which the percent of African American families was high enough to make recruitment economically practical (10% or higher), and in which the percent of families with children living below the poverty line ranged from 10% to 100%. Using these criteria, 259 BGs were identified (115 in Georgia and 144 in Iowa). In both Georgia and Iowa, families were randomly selected from these rosters and contacted to determine their interest in participating in the project. The response rate for the contacted families was 84%. Census tracts included in the FACHS sample were compared with those in Georgia and Iowa to ensure variability and representativeness. There was no significant difference in family income or parental education between the Iowa and Georgia subsamples.

At the first wave, about half of the sample resided in Georgia (n = 422) and the other half in Iowa (n = 467); all of the children were in the fifth grade and averages 10 years of age (*SD* = .63). The vast majority of primary caregivers were women. Given this fact, only women were included in the present study. Mean caregiver age at wave 1 was 37.7 years (*SD* = 8.06), 19.9% had less than a 12th grade education, and 23.3% of families had wo biological parents present. The majority (64.6%) lived in large urban areas, 14.8% lived in the suburbs, and 20.6% lived in rural areas. All of these statistics are roughly comparable to the current demographic profile of Black Americans more generally (Mouzon et al., 2020).

Of the 889 respondents at wave 1, 779 were reinterviewed at wave 2 (1999-2000), 767 at wave 3 (2001-2002), 714 at wave 4 (2004-2005), 689 at wave 5 (2007-2008), and 669 at wave 6 (2010-2011) (75.3 percent of the original sample). In 2015 through 2016, a wave 7 of data collection was completed the included blood draws. The mean age was 29 years. Given the logistics of scheduling home visits of phlebotomists, only members of the sample residing in Georgia, Iowa, or a contiguous state were identified as eligible. After excluding persons who were deceased, incarcerated, or otherwise unreachable, we were left with a pool of 545 samples, 470 (86%) of whom agreed to be interviewed and provide blood. After excluding samples with poor quality and samples excluded because of no amplification, successful assays for mRNA expression was obtained for 386 individuals. In the current study, there were four missing cases for self-reported illness. Analyses are based on 382 respondents (143 men and 239 women).

References

Gibbons, F. X., Gerrard, M., Cleveland, M. J., Wills, T. A., & Brody, G. (2004). Perceived discrimination and substance use in African American parents and their children: a panel study. *Journal of Personality and Social Psychology*, *86*, 517-529.

Mouzon, D. M., Taylor, R. J., & Chatters, L. M. (2020). Gender differences in marriage, romantic involvement, and desire for romantic involvement among older African Americans. *Plos One*, *15*, e0233836.

Simons, R. L., Lei, M. K., Beach, S. R., Brody, G. H., Philibert, R. A., & Gibbons, F. X. (2011). Social environment, genes, and aggression: Evidence supporting the differential susceptibility perspective. *American Sociological Review*, *76*, 883-912.

Simons, R. L., Lei, M. K., Klopach, E., Berg, M., Zhang, Y., & Beach, S. S. (2021). (Re) Setting Epigenetic Clocks: An Important Avenue Whereby Social Conditions Become Biologically Embedded across the Life Course. *Journal of Health and Social Behavior*, *62*, 436-453.

Appendix B. Procedures of mRNA extraction

Participants were asked to provide a blood sample at age 29 (wave 7). After blood was drawn, it was shipped via courier to a laboratory at the University of Iowa and stored until the mRNA analyses were conducted. Samples for quantifying mRNA were collected in a PAXgene tube (Qiagen PAXgene Blood RNA) and frozen after which they were sent to a Rutgers University repository for confidential analysis. The viable samples were processed using the Illumina HumanHT-12 v4 BeadChip. In each case, 200 ng of total RNA was processed according to the protocol supplied by Illumina. All samples were randomized prior to array hybridization using either two or three technical replicates. After background subtraction, raw Illumina probe data were exported using Illumina GenomeStudio v2011.1 software. The microarray data set of 47,323 probes was filtered by removing probes with detection threshold of *p* < .05, and probes with fewer than three beads present were also excluded, leaving 44,846 probes for analysis. Then, robust multi-array average normalized data were log_2_ transformed after quantile normalization and the quality of the microarray images was inspected visually using the ArrayAnalysis quality control pipeline. The results showed that there were no significant batch effects after quantile normalization.

**Appendix C. Items of childhood/adolescent adversity**

**A. Parental hostility** (Adapted from Simons et al., 2019; Conger & Elder, 1994; Coefficient α for this instrument was roughly .80 at each wave)

Response options: 1 = “always,” 2 = “often,” 3 = “sometimes,” and 4 = “never”

1. Get angry at you (Reverse coded)
2. Get so mad at you that he/she broke or threw things (Reverse coded)
3. Shout or yell at you because he/she was mad at you (Reverse coded)
4. Threaten to hurt you physically (Reverse coded)
5. Criticize you or your ideas (Reverse coded)
6. Push, grab, hit, or shove you (Reverse coded)
7. Argue with you whenever you disagreed about something (Reverse coded)
8. Slap or hit you with his/her hands (Reverse coded)
9. Strike you with an object (Reverse coded)
10. Boss you around a lot (Reverse coded)
11. Throw things at you (Reverse coded)
12. Insult or swear at you (Reverse coded)
13. Tell you he/she is right and you are wrong about things (Reverse coded)
14. Give you a lecture about how you should behave (Reverse coded)

**B. Neighborhood crime** (Adapted from Sampson, Raudenbush, Earls, 1997; Coefficient α for this instrument was roughly .75 at each wave)

Response options: 1 = “often,” 2 = “sometimes,” and 3 = “never”

“During the past six months, how often was there...”

1. a fight in your neighborhood in which a weapon like a gun or knife was used?
2. a gang fight?
3. a sexual assault or rape?
4. a robbery or mugging?
5. a murder?

**C. Racial Discrimination** (Adapted from Simons et al., 2019; Whitbeck et al., 2001; Coefficient α for this instrument was roughly .75 at each wave)

Response options: 1 = “never,” 2 = “once or twice,” 3 = “a few times,” and 4 = “several times”

1. How often has someone said something insulting to you just because you are African American?
2. How often has someone ignored you or excluded you from some activity just because you are African American?
3. How often has someone yelled a racial slur or racial insult at you just because you are African American?
4. How often has someone threatened to harm you physically just because you are African American?

**D. Socioeconomic risk** (Adapted by Conger & Elder, 1994; Coefficient α for this instrument was roughly .70 at each wave)

Response options: 1 = “yes,” and 0 = “no”

“During the past 12 months”

1. were you unemployed at any time when you wanted a job?
2. get laid off?
3. suffer a financial loss in business, investments, or property?
4. have a home loan foreclosed on?
5. has your family postponed major household purchase(s) because of financial need?
6. has your family changed residences to save money?
7. has your family changed food shopping or eating habits to save money?
8. has your family reduced driving the car to save money?
9. has you family postponed or delayed paying property tax?

**Reference**

Conger, R. D., Conger, K. J., Elder Jr, G. H., Lorenz, F. O., Simons, R. L., & Whitbeck, L. B. (1992). A family process model of economic hardship and adjustment of early adolescent boys. *Child development*, *63*, 526-541.

Conger, R., & Elder, G. H., Jr. (1994). *Families in troubled times: Adapting to changes in rural America*. New York: Aldine de Gruyter.

Sampson, R. J., Raudenbush, S. W., & Earls, F. (1997). Neighborhoods and violent crime: A multilevel study of collective efficacy. *Science*, *277*, 918-924.

Simons, R. L., Lei, M. K., Beach, S. R., Simons, L. G., Barr, A. B., Gibbons, F. X., & Philibert, R. A. (2019). Testing life course models whereby juvenile and adult adversity combine to influence speed of biological aging. *Journal of health and social behavior*, *60*, 291-308.

Whitbeck, L. B., Hoyt, D. R., McMorris, B. J., Chen, X., & Stubben, J. D. (2001). Perceived discrimination and early substance abuse among American Indian children. *Journal of health and social behavior*, *42*, 405-424.

**Appendix D. Items of adult psychosocial maladjustment**

**A. Depressive and anxiety symptoms (DAS)** (Adapted by Kessler et al., 1994; Carter et al., 2019; Coefficient α for this instrument was roughly .80 at each wave)

Response options: 1 = “yes,” and 0 = “no”

“In the past year, was there ever a two week period”

1. when you felt sad, empty, or depressed most of the day?
2. when you lost interest in things?
3. when you woke up at least two hours before you wanted to?
4. when you couldn't sit still and paced up and down or couldn't keep your hands still when sitting?
5. when you felt worthless nearly every day?
6. when you lost the ability to enjoy having good things happen to you, like winning something or being praised or complimented?
7. when you felt so low that you thought about committing suicide?
8. when you took medication for depression?
9. when you were so depressed or sad that it interfered with your ability to do your job, take care of your house or family, or take care of yourself?

**B. Lack of self-control** (Adapted by Kendall and Wilcox, 1979; Coefficient α for this instrument was roughly .70 at each wave)

Response options: 1 = “not at all true,” 2 = “somewhat true,” 3 = “very true”

1. When you ask a question, you often jump to something else before getting an answer.
2. You have to have everything right away.
3. You have to be reminded several times to do things.
4. You have a lot of accidents.
5. You often have days when you find it difficult to do your school work.
6. You would rather have a small gift today than a large gift tomorrow.
7. You bother other students when they are trying to work.
8. You are easily distracted from your school work.
9. You could be described as careless.
10. You like to switch from one thing to another.
11. If you find that something is really difficult, you get frustrated and quit.

**C. Low self-esteem** (Adapted by Rosenberg et al., 1995; Coefficient α for this instrument was roughly .75 at each wave)

Response options: 1 = “strongly agree,” 2 = “agree,” 3 = “neutral or mixed,” 4 = “disagree,” and 5 = “strongly disagree”

1. I feel I do not have much to be proud of. (Reverse coded)
2. I take a positive attitude toward myself.
3. On the whole, I am satisfied with myself.
4. I certainly feel useless at times. (Reverse coded)
5. I wish I could have more respect for myself. (Reverse coded)
6. At times I think I am no good at all. (Reverse coded)

**Reference**

Carter, S. E., Ong, M. L., Simons, R. L., Gibbons, F. X., Lei, M. K., & Beach, S. R. (2019). The effect of early discrimination on accelerated aging among African Americans. *Health Psychology*, *38*, 1010-1013.

Kendall, P. C., & Wilcox, L. E. (1979). Self-control in children: development of a rating scale. *Journal of Consulting and Clinical psychology*, *47*, 1020-1029.

Kessler, R. C., McGonagle, K. A., Zhao, S., Nelson, C. B., Hughes, M., Eshleman, S., . . . Kendler, K. S. (1994). Lifetime and 12-month prevalence of DSM-III-R psychiatric disorders in the United States: Results from the National Comorbidity Survey. *Archives of General Psychiatry*, *51*, 8-19.

Rosenberg, M. (1965). *Society and the Adolescent Self-Image*. Princeton, NJ: Princeton University Press.

**Appendix E. Items of childhood depression and healthy behavior**

**A. Childhood depression** (Adapted by DISC–IV; Shaffer et al., 1993; Coefficient α for this instrument was .83)

Response options: 1 = “yes,” and 0 = “no”

“In the past year, were there two weeks in a row when you felt”

1. Depressed mood or irritable mood
2. Diminished interest or pleasure
3. Weight loss or weight gain or appetite change
4. Insomnia or hypersomnia
5. Psychomotor agitation or retardation
6. Fatigue or loss of energy
7. Worthlessness or guilt
8. Thinking or concentration problems or indecisiveness
9. Thoughts of death, suicidal ideation, suicide attempt or plan

**B. Healthy diet** (Developed by the FACHS project DISC–IV; Simons et al., 2019)

Response options: 1 = “none,” 2 = “1 or 2 days,” 3 = “3 or 4 days,” 4 =”5 or 6 days,” 5 = “at least once every day,” and 6 = “more than once every day”

1. During the past 7 days, how many times did you eat a whole piece of fruit (for example, an apple, orange or banana) or drink a glass of 100% fruit juice (do not count punch, Kool-Aid, or sports drinks)?
2. During the past 7 days, how many times did you eat vegetables like green salad, carrots or potatoes (do not count French fries, fried potatoes, or potato chips)

Scores on the two items were averaged to form healthy diet at age 29

**C. Exercise** (Developed by the FACHS project DISC–IV; Simons et al., 2019)

Response options: 1 = “0 days,” 2 = “1 or 2 days,” 3 = “3 or 4 days,” 4 =”5 or 6 days,” and 5 = “all 7 days”

1. On how many of the past 7 days did you exercise or participate in physical activity for at least 30 minutes that made you breathe hard (such as basketball, soccer, running, or riding a bicycle hard)?
2. On how many of the past 7 days did you exercise or participate in physical activity for at least 30 minutes that did not make you breathe hard, but was still exercise (such as fast walking, slow bicycling, skating, pushing a lawn mower or doing active household chores)?

**Reference**

Shaffer, D., Schwab-Stone, M., Fisher, P., Cohen, P., Placentini, J., Davies, M., . . . Regier, D. (1993). The diagnostic interview schedule for children-revised version (DISC-R): I. Preparation, field testing, interrater reliability, and acceptability. *Journal of the American Academy of Child & Adolescent Psychiatry*, *32*, 643–650.

Simons, R. L., Lei, M. K., Beach, S. R., Simons, L. G., Barr, A. B., Gibbons, F. X., & Philibert, R. A. (2019). Testing life course models whereby juvenile and adult adversity combine to influence speed of biological aging. *Journal of Health and Social Behavior*, *60*, 291-308.

**Table S1**. Baseline sample (age 10) characteristics for participants retained vs. those lost to attrition

|  | **Retained (n = 382)** |  | **Lost (n = 507)** |  |  |  |
| --- | --- | --- | --- | --- | --- | --- |
|  | Mean  (*SD*) |  | Mean  (*SD*) |  | *t-value* | *p-value* |
| Parental hostility (ages 10) | 22.35  (4.64) |  | 22.06  (4.73) |  | .91 | .36 |
| Neighborhood crime (ages 10) | 6.74  (2.18) |  | 6.63  (2.24) |  | .76 | .45 |
| Racial discrimination (ages 10) | 6.59  (2.53) |  | 6.50  (2.50) |  | .51 | .61 |
| Socioeconomic risk (age 10) | 4.14  (1.11) |  | 4.13  (1.06) |  | .09 | .93 |
| Depression (age 10) | .03  (.11) |  | .03  (.10) |  | .67 | .51 |
| Body mass index (age 10) | 21.73  (5.94) |  | 21.82  (5.87) |  | -.21 | .83 |

*Note*: Variables are measured at the first wave of the study.

**Table S2.** Correlations, means, and standard deviations among study variables (N = 382)

|  | 1 | 2 | 3 | 4 | 5 | 6 | 7 | 8 | 9 | 10 | 11 | 12 | 13 | 14 | 15 |
| --- | --- | --- | --- | --- | --- | --- | --- | --- | --- | --- | --- | --- | --- | --- | --- |
| 1. Parental hostility (ages 10-18) ^a^ | -- |  |  |  |  |  |  |  |  |  |  |  |  |  |  |
| 2. Neighborhood crime (ages 10-18) ^a^ | .12* | -- |  |  |  |  |  |  |  |  |  |  |  |  |  |
| 3. Racial discrimination (ages 10-18) ^a^ | .13* | .10† | -- |  |  |  |  |  |  |  |  |  |  |  |  |
| 4. Socioeconomic risk (ages 10-18) ^a^ | -.03 | .04 | .04 | -- |  |  |  |  |  |  |  |  |  |  |  |
| 5. Low self-esteem (ages 21-25) ^b^ | .13** | .13** | .10† | .12* | -- |  |  |  |  |  |  |  |  |  |  |
| 6. Depressive and anxiety symptoms (ages 21-25) ^b^ | .16** | .06 | .24** | .12* | .51** | -- |  |  |  |  |  |  |  |  |  |
| 7. Lack of self-control (ages 21-25) ^b^ | .11* | -.01 | .14** | .08 | .46** | .43** | -- |  |  |  |  |  |  |  |  |
| 8. Self-reported illness (age 29) | .13** | -.03 | .21** | .04 | .26** | .36** | .27** | -- |  |  |  |  |  |  |  |
| 9. Accelerated biological aging (age 29) | .15** | .17** | .06 | .12** | .14** | .13* | .15** | .13* | -- |  |  |  |  |  |  |
| 10. Depression (ages 10-18) | .18** | .02 | .21** | .10* | .22** | .20** | .13* | .17** | .08 | -- |  |  |  |  |  |
| 11. Depression (age 29) | -.12* | .02 | .08 | .11* | .27** | .31** | .12* | .31** | .06 | .16** | -- |  |  |  |  |
| 12. Males (1 = males) | -.17** | .01 | -.01 | -.08 | -.01 | -.07 | -.03 | -.27** | -.09† | -.11* | -.12* | -- |  |  |  |
| 13. Education (age 29) | .04 | -.13** | .05 | .01 | -.09† | -.06 | .03 | .08 | -.02 | .02 | -.04 | -.07 | -- |  |  |
| 14. Body mass index (ages 10-18) ^b^ | .17** | -.04 | .06 | .08 | .04 | .06 | .06 | .18** | .18** | .11* | .04 | -.14** | .10† | -- |  |
| 15. Health diet (age 29) | .15** | .06 | .12* | .07 | -.09† | -.03 | -.05 | .12* | .03 | .05 | -.01 | -.18** | .19** | .11* | -- |
| 16. Exercise (age 29) | -.02 | .02 | .18** | .01 | -.04 | .01 | .02 | .05 | .01 | .07 | -.02 | .17** | .14** | .01 | .18** |

^†^ *p* ≤ .10; * *p* ≤ .05; ** *p* ≤ .01 (two-tailed tests).

^a^ repeated-measures latent class analysis to identify two subgroups (1 = high-stable/increasing; 0 = low)

^b^ Scores are based on the average of measure across time

**Table S3**. Multiple group comparison between males and females

| Paths | $\chi^{2}$ | *df* | ${\Delta\chi}_{(1)}^{2}$ | *p*-value |
| --- | --- | --- | --- | --- |
| Parental hostility → Adult psychosocial maladjustment |  |  |  |  |
| bs equal for both | 106.19 | 69 |  |  |
| bs free to differ | 103.96 | 68 | 2.23 | .07 |
| Neighborhood crime → Adult psychosocial maladjustment |  |  |  |  |
| bs equal for both | 104.00 | 69 |  |  |
| bs free to differ | 103.96 | 68 | .04 | .59 |
| Racial discrimination → Adult psychosocial maladjustment |  |  |  |  |
| bs equal for both | 105.51 | 69 |  |  |
| bs free to differ | 103.96 | 68 | 1.55 | .22 |
| Socioeconomic risk → Adult psychosocial maladjustment |  |  |  |  |
| bs equal for both | 106.76 | 69 |  |  |
| bs free to differ | 103.96 | 68 | 2.80 | .07 |
| Adult psychosocial maladjustment → Self-reported illness |  |  |  |  |
| bs equal for both | 116.18 | 69 |  |  |
| bs free to differ | 103.96 | 68 | 12.22 | .01 |
| Adult psychosocial maladjustment → Accelerated biological aging |  |  |  |  |
| bs equal for both | 106.28 | 69 |  |  |
| bs free to differ | 103.96 | 68 | 2.32 | .19 |

**Table S4.** Indirect effect models of childhood/adolescent adversity through adult psychosocial maladjustment (indicators: low self-esteem and depressive and anxiety symptoms) on self-reported illness and accelerated biological aging

|  | **Self-reported illness (age 29)** | |  | **Accelerated aging (age 29)** | |
| --- | --- | --- | --- | --- | --- |
|  | Indirect effect | |  | Indirect effect | |
| Model | Coefficient  (95% CI) | Percent  Mediation (*P*_M_) |  | Coefficient  (95% CI) | Percent  Mediation (*P*_M_) |
| **Mediator = Adult psychosocial maladjustment (ages 21-25)** | | | | |  |
| Parental hostility (ages 10-18) | .05*  (.01, .12) | 58.06% |  | .02*  (.01, .06) | 17.31% |
| Neighborhood crime (ages 10-18) | .03  (-.01, .07) | ns |  | .01  (-.01, .04) | ns |
| Racial discrimination (ages 10-18) | .07**  (.02, .14) | 40.37% |  | .02*  (.01. .07) | 75.86% |
| Socioeconomic risk (ages 10-18) | .06**  (.02, .11) | 39.86% |  | .02*  (.01. .06) | 15.00% |

*Note*: Indirect effect represents the indirect effects of the predictors on self-reported illness and accelerated biological aging via the latent variable of adult psychosocial maladjustment (indicators: low self-esteem and depressive and anxiety symptoms). Precent meditation (*P*_M_) is (|Indirect effect| ÷ |Total effect|) × 100. Coefficients are standardized. 95% CI is 95% confidence interval.

***p*≤.01; **p*≤.05, †*p*<.10 (two-tailed tests).
